# What impact do questionnaire length and monetary incentives have on mailed health psychology survey response?

**DOI:** 10.1111/bjhp.12239

**Published:** 2017-04-19

**Authors:** Kathryn A. Robb, Lauren Gatting, Jane Wardle

**Affiliations:** ^1^ Institute of Health and Wellbeing General Practice and Primary Care University of Glasgow UK; ^2^ Health Behaviour Research Centre Department of Epidemiology and Public Health UCL London UK

**Keywords:** cancer screening, colorectal, incentives, length, mailed surveys, questionnaires, response rates, UK

## Abstract

**Objectives:**

Response rates to health‐related surveys are declining. This study tested two strategies to improve the response rate to a health psychology survey mailed through English general practices: (1) sending a shortened questionnaire and (2) offering a monetary incentive to return a completed questionnaire.

**Design:**

Randomized controlled trial.

**Methods:**

Adults (*n* = 4,241) aged 45–59 years, from four General Practices in South‐East England, were mailed a survey on attitudes towards bowel cancer screening. Using a 2 × 4 factorial design, participants were randomized to receive a ‘short’ (four A4 pages) or a ‘long’ (seven A4 pages) questionnaire, and one of four monetary incentives to return a completed questionnaire – (1) no monetary incentive, (2) £2.50 shop voucher, (3) £5.00 shop voucher, and (4) inclusion in a £250 shop voucher prize draw. Age, gender, and area‐level deprivation were obtained from the General Practices.

**Results:**

The overall response rate was 41% (*n* = 1,589). Response to the ‘short’ questionnaire (42%) was not significantly different from the ‘long’ questionnaire (40%). The £2.50 incentive (43%) significantly improved response rates in univariate analyses, and remained significant after controlling for age, gender, area‐level deprivation, and questionnaire length. The £5.00 (42%) and £250 prize draw (41%) incentives had no significant impact on response rates compared to no incentive (38%).

**Conclusions:**

A small monetary incentive (£2.50) may slightly increase response to a mailed health psychology survey. The length of the questionnaire (four pages vs. seven pages) did not influence response. Although frequently used, entry into a prize draw did not increase response. Achieving representative samples remains a challenge for health psychology.

**Statement of contribution**

***What is already known on this subject***

- Response rates to mailed questionnaires continue to decline, threatening the representativeness of data.
- Prize draw incentives are frequently used but there is little evidence to support their efficacy.
- Research on interactions between incentives, questionnaire length, and demographics is lacking.

***What does this study add***

- Contrary to previous findings, questionnaire length did not influence response rate.
- A £2.50 incentive increased response, while incentives of £5.00 and a £250 prize draw did not.
- Achieving representative samples to questionnaires remains a challenge for health psychology.

## Background

The majority of data gathered for health psychology research involve the use of questionnaires or surveys (Marks, Murray, Evans, & Estacio, 2015). However, the representativeness of the data generated from questionnaires is threatened by the persistent decline in response rates (Fulton, 2016; Tourangeau & Plewes, 2013). Low response rates have serious implications for the generalizability of the results of a survey and for the accuracy of any estimates made (Groves & Peytcheva, 2008). We know from previous work that lower socio‐economic groups are less likely to participate in research (McCaffery, Wardle, Nadel, & Atkin, 2002), and this will further undermine the generalizability of surveys. It is important that we identify methods to increase response, while also improving the distribution of responses, to be more representative of the population.

Edwards *et al*.'s (2009) Cochrane review identified that using monetary incentives nearly doubled response compared to no incentive, OR: 1.87 (CI 1.73–2.03), and many studies have shown that only a small amount of money (e.g., £5 or $1) is required to have a significant effect on response (Chan, Tse, Day, Tong, & Suen, 2003; Ulrich *et al*., 2005; VanGeest, Wynia, Cummins, & Wilson, 2001). Edwards *et al*.'s (2009) review also found that a bigger monetary incentive was more effective, OR = 1.26 (CI 1.14–1.39). However, the shape and strength of the relationship between incentive size and response is less clear, from the review with some studies demonstrating a positive correlation (Trussell & Lavrakas, 2004) and others not finding any significant correlation (Chan *et al*., 2003; VanGeest *et al*., 2001). Shortening the questionnaire length has also been shown to be effective in increasing response (Edwards *et al*., 2009; Singer & Ye, 2013).

However, much of the research into increasing mailed survey response has been conducted in the United States, with only a few studies carried out in the United Kingdom (Cartwright, 1986; Deehan, Templeton, Taylor, Drummond, & Strang, 1997; Kenyon *et al*., 2005; Roberts, Wilson, Roalfe, & Bridge, 2004). There are cultural differences between the United States and the United Kingdom; most notable for this research is that the United Kingdom has a national health service that is free at the point of delivery. Response to a survey sent within the UK health care context may therefore be quite different to those conducted in the United States. Also, none of the studies examined response to a health psychology questionnaire among a non‐patient population.

Many health surveys use prize draws as a cheaper way to incentivize participation as this requires less money than paying each participant individually (Bowling, 2014). However, most studies looking at the effectiveness of inclusion in a prize draw on response have found this type of incentive to be no more effective than receiving no incentive (Edwards *et al*., 2009). However, the effectiveness of prize draw incentives may be mediated by participant characteristics (Drummond, O'Leary, O'Neill, Burns, & Sharp, 2014; Spry *et al*., 1989).

Research on survey response has yet to build a clear picture on the interaction between demographic characteristics and the combined use of incentives and questionnaire length. There is variability in incentive effectiveness across different populations and demographic groups. For example, Boulianne (2013) found gender to interact with amount of incentive ($5 vs. $10) in increasing likelihood of responding to a web‐based survey about their general interests and hobbies, such that women were more likely to respond to $5 compared to $10. Other research has found a differential effect of incentive on response across levels of poverty. In a survey experiment using the US Survey of Income and Program Participation, Mack, Huggins, Keathley, and Sundukchi (1998) found $20, over no incentive, to be particularly effective in increasing response for participants who were living in greater poverty.

### The present study

Achieving representative samples to questionnaires remains a challenge for health psychology but is under‐researched. Research into interaction effects between monetary incentives, questionnaire length, and participant demographic characteristics is lacking. Prize draws are frequently used but there is little evidence to support their efficacy. The current randomized trial was undertaken to compare the effect of four different incentive types and two questionnaire lengths on the response rates of a primary care population to a survey about bowel cancer screening. The aim was also to investigate any interaction effects between, questionnaire length, incentive type, and participant demographic characteristics.

The following hypotheses were made:

1. A four‐page questionnaire will produce a higher response rate than a seven‐page questionnaire.
2. A monetary incentive of £2.50 will produce a higher response rate than no incentive.
3. A monetary incentive of £5.00 will produce a higher response rate than £2.50.
4. A monetary prize draw incentive will produce a higher response rate than no incentive.
5. No predictions were made about the interaction effect of demographic factors on the effectiveness of each of these administration techniques, due to the paucity of previous research in the area.

## Method

### Sample

**Figure 1:**
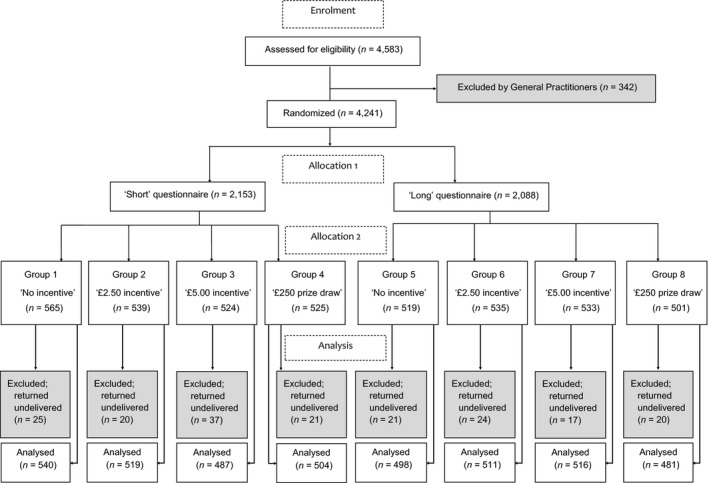
Study design.

Eligible participants were aged 45–59 years, registered with one of four General Practices in South‐East England (*n* = 4,583). This age group was selected because it is approaching the eligible age (60 years) for bowel screening in England. To ensure a socio‐economically and ethnically diverse sample, the four General Practices were selected based on their postcodes which were linked to area‐level deprivation from the Index of Multiple Deprivation (IMD; 2007) and ethnicity data from the 2001 Census. Frequency plots showed that the range of IMD scores within our data was broadly representative of 2007 IMD data for all of England (Communities and Local Government, 2007). General practitioners (GPs) were asked to exclude anyone known to have a recent diagnosis of cancer or those who were considered to be ‘vulnerable’ (e.g., learning disabled, cognitively impaired). This resulted in the exclusion of 342 (8%) people, leaving 4,241 potential participants, of which 1,996 (47%) were women. Participants were randomized by General Practice to one of eight groups (Figure 1).

### Procedure

The remaining 4,241 potential participants were mailed a letter from their GP inviting them to participate in a ‘health and bowel cancer screening survey’, the study questionnaire, and a freepost reply envelope. Participants were randomized to receive one of two questionnaires; the ‘short’ questionnaire (four pages) was sent to 2,153 potential participants, and the ‘long’ questionnaire (seven pages) was sent to 2,088 participants. Participants were also randomized to one of four incentive groups as a ‘thank you for taking the time to fill out the questionnaire’ if they returned a completed questionnaire: (1) no incentive (*n* = 1084); (2) a £2.50 voucher (*n* = 1074); (3) a £5.00 voucher (*n* = 1057); or (4) the chance to win a £250 voucher in a prize draw (*n* = 1026). Participants were able to choose the shop their voucher could be used in from one of three popular shops in the United Kingdom – Boots, Tesco, or Argos. Table 1 describes the participant demographic characteristics across the eight experimental groups. Statistical tests confirmed that none of these demographics significantly differed across the experimental groups.

**Table 1:** Participant demographic characteristics

	Short questionnaire				Long questionnaire				Significance
	No incentive	£2.50 incentive	£5.00 incentive	£250 Prize draw incentive	No incentive	£2.50 incentive	£5.00 incentive	£250 Prize draw incentive	
Age years Mean (*SD*)	51.3 (4.08)	51.2 (4.16)	51.0 (4.06)	51.0 (4.22)	50.8 (4.14)	51.2 (4.22)	50.7 (4.12)	51.3 (4.27)	*F* (3872) = 1.61, *p *=* *.129
Gender									
Male (*n* = 2245)	13.4 (269)	13.5 (270)	12.1 (243)	11.8 (236)	11.9 (239)	12.1 (243)	13.3 (267)	12.0 (240)	
Female (*n* = 1996)	13.4 (250)	12.4 (231)	11.8 (220)	13.4 (250)	12.3 (229)	13.0 (243)	12.0 (223)	11.7 (219)	χ^2^ (7, 3872) = 5.20, *p *=* *.635
IMD 2007 Scores Mean (*SD*)	16.1 (13.5)	14.3 (13.8)	16.1 (15.1)	15.1 (15.0)	15.7 (14.5)	15.2 (14.5)	14.8 (13.6)	14.9 (14.1)	*F* (3870) = 1.00, *p *=* *.430

% (*n*) unless otherwise stated; *SD* = standard deviation.

Participants from the same household were sent the same version of the survey and incentive. Non‐responders were sent a reminder questionnaire after approximately 2 weeks. All letters included ‘return to sender if undelivered’ instructions on the reverse of the envelope. All questionnaires were mailed between June and December 2009. Ethical approval was granted by the NRES London Bridge Committee.

### Measures

#### ‘Short’ questionnaire

The short questionnaire contained four pages of questions. The questionnaire covered intentions to screen (two items), having previously done an FOB test (one item), six items from the State‐Trait Anxiety Inventory (STAI; Marteau & Bekker, 1992), seven items from the Behavioural Inhibition System (BIS; Carver & White, 1994), 24 items from the Rational‐Experiential Index (REI; Norris, Pacini, & Epstein, 1998), preference for information in the form of words compared to percentages (three items), demographic characteristics (age, gender, ethnic group, employment status, and living arrangements), experience of cancer themselves and among family and friends (three items), and whether they wanted to receive the results of the study and participate in future research (three items).

#### ‘Long’ questionnaire

The long questionnaire contained seven pages of questions and included the same information and questions that were given in the short questionnaire. The long questionnaire also included questions on health and lifestyle (10 items), 14 items on coping strategies taken from Brief COPE inventory (Carver, 1997), and 16 items from the Miller Behavioural Style Scale (MBSS; Steptoe, 1989), and 21 items on attitudes towards bowel cancer screening adapted from several questionnaires (Lipkus & Klein, 2006; Miles, Voorwinden, Chapman, & Wardle, 2008; Tiro *et al*., 2005), and questions on perceived risk of bowel cancer (four items; Weinstein *et al*., 2007).

Gender data were also obtained from the General Practices to check that the intended recipient had completed the questionnaire. Socio‐economic deprivation was assessed by IMD (2007) score from participants’ home addresses.

### Data analysis

SPSS Statistics for Windows, version 22.0, was used to analyse the data. Chi‐square tests were used to examine response rates between groups in terms of age (45–49 years; 50–54 years; 55–59 years), gender (female; male), IMD score 2007 quartiles, incentive (no incentive; £2.50; £5.00; £250 prize draw), and questionnaire length (short; long). Multiple‐variable logistic regressions were used to determine the independent influence on response rate of age, gender, IMD score, incentive group, and questionnaire length. Requests for access to the original data should be addressed to the corresponding author.

## Results

### Overall response

Of the 4,241 questionnaires mailed out, 185 (4%) were returned as undelivered. A further 184 (4.3%) of respondents were excluded from analysis because the self‐reported gender did not match the GP data, suggesting the intended recipient may not have completed the questionnaire. 156 questionnaires were returned blank and these were included as ‘no response’. There was a 41% response rate (*n* = 1,589/4,241–369).

### Respondent characteristics

Older participants were more likely to return a questionnaire than younger participants, with those aged 55–59 more likely to respond (45.5%) compared to participants in the 45–49 (39.6%) and 50–54 (39.4%) age groups (Table 2). More women returned the questionnaire (45.9%) than men (36.5%). Participants from more affluent neighbourhoods were significantly more likely to return a completed questionnaire (IMD quartile 3 = 43.2%; IMD quartile 4 = 45.8%) compared to those from more deprived neighbourhoods (IMD quartile 1 = 39.1%; IMD quartile 2 = 35.9%).

**Table 2:** Response rates by demographic characteristics, type of incentive, and questionnaire length

	No response or returned blank % (*n*) (*n* = 2,283)	Replied % (*n*) (*n* = 1,589)	Significance
Age (years)			
45–49 (*n* = 1,712)	60.4 (976)	39.6 (640)	
50–54 (*n* = 1,318)	60.6 (771)	39.4 (501)	
55–59 (*n* = 1,027)	54.5 (536)	45.5 (448)	χ² (1, 3872) = 7.51, *p* = .006
Gender			
Male (*n* = 2,148)	63.5 (1274)	36.5 (733)	
Female (*n* = 1,909)	54.1 (1009)	45.9 (856)	χ² (1, 3872) = 35.12, *p* < .001
IMD 2007 quartiles			
1 (deprived) (*n* = 1,044)	60.9 (613)	39.1 (394)	
2 (*n* = 987)	64.1 (606)	35.9 (340)	
3 (*n* = 1,018)	54.0 (546)	43.2 (416)	
4 (affluent) (*n* = 1,006)	53.8 (518)	45.8 (437)	χ² (1, 3870) = 14.55, *p* < .001
Type of incentive			
No incentive (*n* = 1,033)	61.8 (610)	38.2 (377)	
£2.50 (*n* = 1,031)	56.7 (560)	43.3 (427)	
£5.00 (*n* = 1,007)	58.0 (553)	42.0 (400)	
£250 prize draw (*n* = 986)	59.3 (560)	40.7 (385)	χ² (3, 3872) = 5.69, *p* = .128
Questionnaire length			
Long (*n* = 1,985)	59.8 (1138)	40.2 (765)	
Short (*n* = 2,072)	58.2 (1145)	41.8 (824)	χ² (1, 3872) = 1.09, *p* = .297

IMD = Index of Multiple Deprivation; *n* = number.

### Questionnaire length

Response rates did not differ between the ‘long’ questionnaire (40.2%) and the ‘short’ questionnaire (41.8%, Table 2). There were no significant differences in response to the different questionnaire lengths by gender or level of deprivation. However, participants aged 45–49 were more likely to respond to a shorter questionnaire, short = 42.9%,long = 36.4%; χ² (1, 1616) = 7.06, *p* = .008. The other age groups did not significantly differ in their response between the two questionnaire lengths.

### Impact of incentives

Response rates did not differ significantly between people offered an incentive of £2.50 (43.3%), £5.00 (42%), entry into a £250 prize draw (40.7%), or no incentive (38.2%; Table 2). However, when the £2.50 and £5.00 monetary incentives were combined and compared to no monetary incentive, the group offered a monetary incentive had a higher response rate (42.6%) than those offered no incentive, 38.2%; χ² (1, 2927) = 5.31, *p *=* *.021.

There were no significant differences in response to the different incentives by age or gender. However, those who received a £5.00 incentive significantly differed in their response by neighbourhood deprivation, such that those from more affluent neighbourhoods were more likely to respond, IMD quartile 1 (deprived) = 39.3%, IMD quartile 2 = 35.4%, IMD quartile 3 = 45.3%, IMD quartile 4 (affluent) = 52.1%; χ² (1, 953)  = 6.00, *p *=* *.028.

### Multiple‐variable logistic regressions

Logistic regressions were used to predict response rate (no response; response) using each predictor variable. In line with the univariable analyses, the multivariable analyses found a main effect of age, gender, and IMD score, with respondents more likely to be older, female, and from an affluent neighbourhood (Table 3). Questionnaire length was not related to response. Receiving a £2.50 incentive significantly increased the odds of returning a questionnaire compared to no incentive. Effect of incentive remained similar after controlling for age, gender, and IMD score, suggesting that there were no main interaction effects.

**Table 3:** Logistic regression of response (0 = no response or blank; 1 = response)

	Univariable logistic regression	Multivariable logistic regression	
	Response OR (95% CI)	Response OR (95% CI)	Response OR (95% CI)
Age (years)			
45–49	1.00	–	1.00
50–54	0.99 (0.85, 1.15)		0.98 (0.85, 1.14)
55–59	1.28 (1.09, 1.50)*		1.25 (1.06, 1.46)*
Gender			
Male	1.00	–	1.00
Female	1.48 (1.29, 1.68)**		1.47 (1.29, 1.67)**
IMD 2007 quartiles			
1 (deprived)	1.00	–	1.00
2	0.87 (0.73, 1.05)		0.86 (0.71, 1.03)
3	1.19 (0.99, 1.42)		1.16 (0.98, 1.39)
4 (affluent)	1.31 (1.10, 1.57)*		1.29 (1.07, 1.54)*
Incentive			
No incentive	1.00	1.00	1.00
£2.50	1.23 (1.03, 1.48)*	1.24 (1.03, 1.48)*	1.21 (1.01, 1.46)*
£5.00	1.17 (0.98, 1.40)	1.17 (0.98, 1.41)	1.18 (0.98, 1.41)
£250 prize draw	1.11 (0.93, 1.34)	1.11 (0.93, 1.34)	1.08 (0.90, 1.30)
Questionnaire length			
Long	1.00	1.00	1.00
Short	1.07 (0.94, 1.22)	1.07 (0.95, 1.22)	1.08 (0.95, 1.23)

IMD = Index of Multiple Deprivation; OR = odds ratio; CI = confidence interval.

*p < .05; **p < .001.

## Discussion

Declining response rates are threatening the quality of data collected by health surveys. Previous meta‐analyses have identified small monetary incentives and shortening the length of questionnaires to be cost‐effective methods for increasing response rates (Edwards *et al*., 2009; Singer & Ye, 2013). However, research is yet to draw any clear conclusion about the combined influence of monetary incentives, questionnaire length, and individual differences on survey response.

### Main findings

Contrary to previous findings, the current study found that questionnaire length did not influence response rates (Edwards *et al*., 2009). It is possible that the two lengths of questionnaire used in the current study (four pages vs. seven pages) did not differ enough to produce a significant difference in response. It would be useful to compare a variety of lengths to investigate the shape and strength of the relationship between questionnaire length and response.

The current study found that receiving a £2.50 incentive increased response compared to no incentive, supporting our first hypothesis and previous research showing that use of even a small monetary incentive in mailed surveys is more effective than not using an incentive (Edwards *et al*., 2009). Receiving a £5.00 incentive did not have a significant effect on response compared to no incentive, demonstrating that increase in incentive value and response do not share a simple positive linear relationship. The results may be usefully interpreted with reference to Social Exchange Theory (Dillman, Smyth, & Christian, 2009), which suggests that an individual's feeling of social obligation to complete a task is dampened when the amount of money given for completing that task approaches actual payment. In the present study, the offer of a £5.00 voucher may have been perceived as reaching a value equivalent to a payment for completing a four‐ to seven‐page questionnaire, thus reducing the intrinsic motivation of social obligation. Of note, people from more affluent neighbourhoods were significantly more likely to respond to the £5.00 incentive, while there was no difference in response for the £2.50 incentive across levels of deprivation. This suggests that different socio‐economic groups may have different perceptions about what constitutes a payment rather than an incentive.

A prize draw incentive was found to be no more effective than no incentive in increasing response regardless of length of questionnaire. This is in line with previous findings, with only one of 11 studies that looked at prize draw incentives in the Edwards *et al*.'s (2009) review finding a significant effect of prize draw incentives on response.

The only previous study simultaneously considering questionnaire length and prize draw incentive found prize draw incentive to be more effective with a shorter questionnaire (Spry *et al*., 1989). It would be useful to carry out further research in which survey length and size of prize draw incentive are experimentally manipulated to determine whether there is an interaction between these two factors.

In our study, older people, women, and those living in less deprived areas were most likely to respond, and these effects remained significant in multi variable analysis. The sample in this study was potentially limited in that it had only a small age range and used a measure of deprivation based on neighbourhood postcode that could be seen as less accurate than an individual measure (Fischbacher, 2014). However, Roberts *et al*. (2004) also found age, gender, and level of deprivation to influence response. In their sample, ages ranged from 18 to 100 years and deprivation was measured using the Townsend score. Affluent individuals and women are consistently found to be most likely to participate in surveys (Smith, 2008). This seriously impacts the generalizability of the results of any survey as they will not be representative of the general population (Groves & Peytcheva, 2008). It is imperative that future research into survey administration considers how to increase response from underrepresented groups based on demographic characteristics.

The findings from our research on survey response can also be considered within the broader context of incentivizing health behaviour. So far, research has found monetary incentives to be effective in improving health behaviours for changing small and discrete behaviours that are time‐limited (Lynagh, Sanson‐Fisher, & Bonevski, 2013). However, research has also shown that improving intrinsic motivations, such as altruism or self‐worth, can be more effective in changing behaviour than in increasing extrinsic motivation, such as monetary incentives (Sutherland, Leatherman & Christianson, 2008). Curry, Wagner, and Grothaus (1991) found that patients who received personal feedback (intrinsic motivator) alongside smoking cessation self‐help material were twice as likely to have abstained at 12 months compared to patients who had received a monetary incentive (extrinsic motivator), both with and without the personal feedback. People from lower socio‐economic backgrounds have been shown to be more responsive to monetary incentives particularly when the amount is perceived to address an economic barrier (Lagarde, Haines, & Palmer, 2007). It has been suggested that, under this circumstance, receiving money does not ‘crowed out’ the patients’ intrinsic motivations because the money is perceived as an enabler (Frey & Jegen, 2001). It could be argued that in the current study the £5.00 incentive ‘crowded out’ intrinsic motivation for people in the deprived group but not for people in the affluent group. Previous health behaviour change studies have found incentives higher than £5.00 to be effective in changing behaviour; for example, effective incentive sizes for smoking cessation trials have ranged from US$20 to US$750 (Volpp *et al*., 2006, 2009). Therefore, £5.00 is a comparatively low threshold for ‘crowding out’ to be experienced. It is likely that participants considered the task of completing and posting a survey to be undemanding and as having few economic barriers meaning payment for this task would equate to a much smaller amount.

### Implications

This study contributes to an under‐researched topic in health research in the United Kingdom. Our findings suggest using a minimal amount of money (£2.50) to incentivize response may be justifiable to increase response rates without significantly impacting the demographic composition of the final sample. In contrast, our study suggests using a £5.00 incentive may disproportionately increase response rates of people from affluent neighbourhoods, leading to the further underrepresentation of people from deprived neighbourhoods in the sample.

Prize draws may seem an attractive option for increasing response rate for a mailed survey because it is cheaper than giving every respondent monetary payment for taking part. However, the results of this study support growing research evidence that this style of incentive is ineffective (Edwards *et al*., 2009). Young *et al*. (2015) have most recently looked at conditional versus unconditional incentives as a different way of conceptualizing pre‐payment and post‐payment, finding unconditional incentives to be much more effective.

### Limitations

The topics of personal health practices, beliefs, and bowel cancer screening may have influenced response to this study (Cantor, O'Hare, & O'Connor, 2007; Van Kenhove, Wijnen, & De Wulf, 2002). For example, disgust is an emotion commonly reported in association with bowel screening that may have influenced response (Chambers, O'Carroll, Brownlee, Libby, & Steele, 2016; O'Carroll, Chambers, Brownlee, Libby, & Steele, 2015; O'Sullivan & Orbell, 2004). There is little to suggest that completing a health questionnaire with a portion of the questions related to bowel cancer screening will evoke a strong disgust reaction or that this will influence the effect of incentive or questionnaire length on response in a certain way. However, it is important to consider the topic of the survey when comparing the results to other studies.

It was unusual that the chi‐square test did not show a significant difference in response across incentive groups, while the univariable logistic regression did. This brings into question whether the analyses were appropriately powered. Post‐hoc power analyses determined that the chi‐square test was slightly underpowered (70.6%), while the logistic regression was above 80 (89.1%), which is considered high. This suggests that the £2.50 incentive significantly increased the odds of responding.

The age range of the sample was limited to 45–59 years. However, the findings remain important because surveys are becoming increasingly targeted to particular demographic characteristics, such as specific age groups. Further research could consider the impact of incentives on response for other age groups. From our study, we are unable to determine the extent to which non‐contact (i.e., the intended recipient does not receive the survey) accounted for non‐response. Non‐contact due to situational factors, such as the respondent having moved with no forwarding address, has been found to be higher for lower socio‐economic groups (Goodman & Gatward, 2008). This may lead to exaggerated results for non‐response in this group as these types of non‐response will not be affected by the experimental conditions. Similarly, there was no way of knowing whether intended recipients were the people sending back the surveys, although this was considered to some extent through comparing gender reported by participants with gender given by the GPs. This showed that at least 4% of responses were being completed by the wrong person.

Additionally, we did not investigate whether quality of response (i.e., how much effort participants put into completing the questionnaire) differed between the different methods of survey administration. Currently, there is no empirical way of determining the extent to which a response has been well considered, unless through self‐report.

### Conclusion

The present study found that the length of the questionnaire (four pages vs. seven pages) did not influence response. An incentive of £2.50 slightly increased response but the offer of £5.00 or entry into a £250 prize draw did not influence response. Through looking at the interaction between demographic characteristics and incentive type, the current study was able to demonstrate that using monetary incentives does not necessarily improve the quality of mailed survey data, given that increase in response does not equate to a more representative sample or better quality of response.

The study found that a larger incentive of £5.00 significantly increased response if people were from an affluent neighbourhood but not if they were from deprived neighbourhoods. This suggests that monetary incentives of a certain amount could work to further marginalize people with lower socio‐economic status, which brings into question the appropriateness of using monetary incentives over other methods of increasing survey engagement. It is also clear from this result that, to identify ways of achieving greater and more representative survey uptake in the future, researchers need to consider what impact key qualities of their survey, and the ways in which it is being administered, are having on response. Investigating the potential interacting effect of survey properties and different demographic characteristics on response will also be essential in this endeavour. Achieving representative samples to questionnaires remains a challenge for health psychology research.

## Conflict of interest

All authors declare no conflict of interest.
